# Assessment of trunk flexion in arm reaching tasks with electromyography and smartphone accelerometry in healthy human subjects

**DOI:** 10.1038/s41598-021-84789-3

**Published:** 2021-03-08

**Authors:** Yolanda Castillo-Escario, Hatice Kumru, Josep Valls-Solé, Loreto García-Alen, Joan Vidal, Raimon Jané

**Affiliations:** 1 Institute for Bioengineering of Catalonia, Barcelona Institute of Science and Technology, 08028 Barcelona, Spain; 2 Department of Automatic Control, Universitat Politècnica de Catalunya-Barcelona Tech (UPC), 08028 Barcelona, Spain; 3 Centro de Investigación Biomédica en Red de Bioingeniería, Biomateriales y Nanomedicina, 28029 Madrid, Spain; 4 Fundación Institut Guttmann, Institut Universitari de Neurorehabilitació Adscrit a la UAB, 08916 Badalona, Spain; 5 Universitat Autònoma de Barcelona, Bellaterra, 08193 Barcelona, Spain; 6 Fundació Institut d’Investigació en Ciències de la Salut Germans Trias i Pujol, 08916 Badalona, Spain; 7 Institut d’Investigació August Pi i Sunyer (IDIBAPS), 08036 Barcelona, Spain

**Keywords:** Biomedical engineering, Electromyography - EMG, Motor control, Sensorimotor processing

## Abstract

Trunk stability is essential to maintain upright posture and support functional movements. In this study, we aimed to characterize the muscle activity and movement patterns of trunk flexion during an arm reaching task in sitting healthy subjects and investigate whether trunk stability is affected by a startling acoustic stimulus (SAS). For these purposes, we calculated the electromyographic (EMG) onset latencies and amplitude parameters in 8 trunk, neck, and shoulder muscles, and the tilt angle and movement features from smartphone accelerometer signals recorded during trunk bending in 33 healthy volunteers. Two-way repeated measures ANOVAs were applied to examine the effects of SAS and target distance (15 cm vs 30 cm). We found that SAS markedly reduced the response time and EMG onset latencies of all muscles, without changing neither movement duration nor muscle recruitment pattern. Longer durations, higher tilt angles, and higher EMG amplitudes were observed at 30 cm compared to 15 cm. The accelerometer signals had a higher frequency content in SAS trials, suggesting reduced movement control. The proposed measures have helped to establish the trunk flexion pattern in arm reaching in healthy subjects, which could be useful for future objective assessment of trunk stability in patients with neurological affections.

## Introduction

Trunk stability, defined as the ability to control the position and motion of the trunk, is essential for maintenance of body balance in the upright position under both static and dynamic conditions^1^. This requires control of neck, shoulder, trunk, and pelvic muscles for compensation of external and internal perturbations during loading and movement^2^. These control commands, which include anticipatory, accompanying, and compensatory postural adjustments, are in most instances part of the motor plan, i.e., the package of instructions contained in the descending volleys issued to perform a given action^3^. Sensorimotor integration in the central nervous system (CNS) provides for the muscle recruitment patterns needed to achieve an effective task-specific postural control^4^.

The muscles involved in trunk stabilization during a voluntary movement must work cooperatively, with different contributions depending on the task being performed. One such task, frequently performed in our daily routines, is arm reaching for a relatively distant object, requiring some degree of body tilting, while sitting. In these cases, trunk muscles may be activated with a double purpose: they contribute to task execution by approaching the body to the target and to the postural adjustments needed to maintain the body balance and prevent destabilization^5,6^. However, postural disorders and defective control of trunk muscles are frequently observed in individuals with neurological injuries, such as stroke^7–11^ or spinal cord injury (SCI)^12–14^. In these patients, impaired trunk stability is by itself a major cause of dependency and motor disability, as it limits not only the maintenance of static posture and trunk movements but also functional limb movements, such as reaching tasks and gait^15,16^.

Numerous clinical tests have been proposed to assess sitting and standing balance after stroke or SCI, such as the Trunk Control Test^17,18^, the Trunk Impairment Scale^19^, and the Modified Functional Reach Test^20,21^. A limitation of these tests is their qualitative nature, which makes them prone to subjective bias. Quantitative measures of trunk performance have also been described, which are obtained using biomedical instrumentation, including force plates to measure changes in the centre of pressure and postural sway^22–24^, surface electromyography (EMG) to record muscle activation patterns^25,26^, dynamometers to measure trunk strength^14^, inclinometers to measure the lumbar range of motion^10^, and motion capture systems to obtain kinematic data^27^. Regarding movement analysis, smartphone sensors, such as the gyroscope and accelerometer, have been proposed in other applications as simple cost-effective tools to analyse body movements. For instance, the accelerometer of a smartphone fixed on the thorax may help to identify physical movements^28^, investigate falls^29^, or monitor sleep position overnight^30^. In this study, we considered to use a smartphone accelerometer to monitor trunk tilting during an arm reaching task while sitting.

The potentially destabilizing effect of the described task could be increased if a startling auditory stimulus (SAS) is concomitantly applied. The startle response is a sudden involuntary defensive response to unexpected or threatening stimuli. This basic physiological reaction, which is common to all mammals, has a protective function against injury^31^. Nevertheless, it has been shown that when a SAS is applied together with the imperative signal in a simple reaction time task experiment, i.e., when subjects are fully prepared for task execution, reaction time is significantly shortened, a phenomenon known as StartReact^32,33^. The StartReact effect has been described in simple tasks, such as reaching arm movements^31,33^, ballistic wrist flexion and extension^32^, finger movements^34^, or head flexion and rotation^35^. It has also been reported in complex manoeuvres, such as sit-to-stand^36^, step initiation^37^, obstacle avoidance during walking^38^, self-induced falls^39^, and others. In these manoeuvres, the latency shortening in the activation of muscles engaged in postural adjustments is proportional to the shortening occurring in prime movers.

Although the StartReact phenomenon has long been studied in the literature, there are very few works on trunk muscles or trunk movements^36,40^, and none on an arm reaching task requiring body tilting. In performing such task, many trunk muscles would be engaged in postural adjustments to avoid trunk destabilization. There is a possible conflict between the generation of a reflex response to SAS, which would lead to a protective pattern of activation of trunk muscles, and the execution of the voluntary command, accelerated by the SAS, which would lead to a recruitment in accordance with the motor program. Testing the addition of a SAS to our task has a twofold purpose: to investigate the response of axial muscles engaged in postural control in a StartReact paradigm, and to serve as an unexpected stimulus to unbalance the body and thus examine the compensatory reaction of trunk muscles. Based on the previous literature, we hypothesize that a SAS will shorten the response time, but it is unclear if this will also induce a change in the recruitment pattern of postural muscles with potential consequences in trunk stabilization.

The main objectives of this study are to establish the patterns of trunk muscles activation during trunk flexion in an arm reaching task in healthy subjects and to determine whether target distance and/or SAS application in the context of a StartReact paradigm has any destabilizing effects. For these purposes, we analysed EMG and smartphone accelerometer signals recorded during forward and backward trunk bending in sitting healthy volunteers reaching for a switch button. This approach will contribute to characterize trunk movement patterns in healthy subjects. The task we selected was in sitting position, so that the test could eventually be applied to wheelchair-bound hemiplegic, paraplegic, and tetraplegic patients, in whom the disequilibrium related to body tilting is a relevant handicap and an important goal of rehabilitation.

## Methods

### Participants

Thirty-three healthy subjects were enrolled in this study (17 males and 16 females, aged 20 to 61 years, mean age 38 years). All but one were self-reported right-handers, and all of them were free from neurological and musculoskeletal disorders.

The study was conducted at the Institut Guttmann, Badalona, Spain. The protocol was approved by the Ethics Committee of the Institut Guttmann, and written informed consent was obtained from all the participants prior to the experiment. Research was carried out in accordance with the standards of the Declaration of Helsinki.

### Experimental protocol

**Figure 1:**
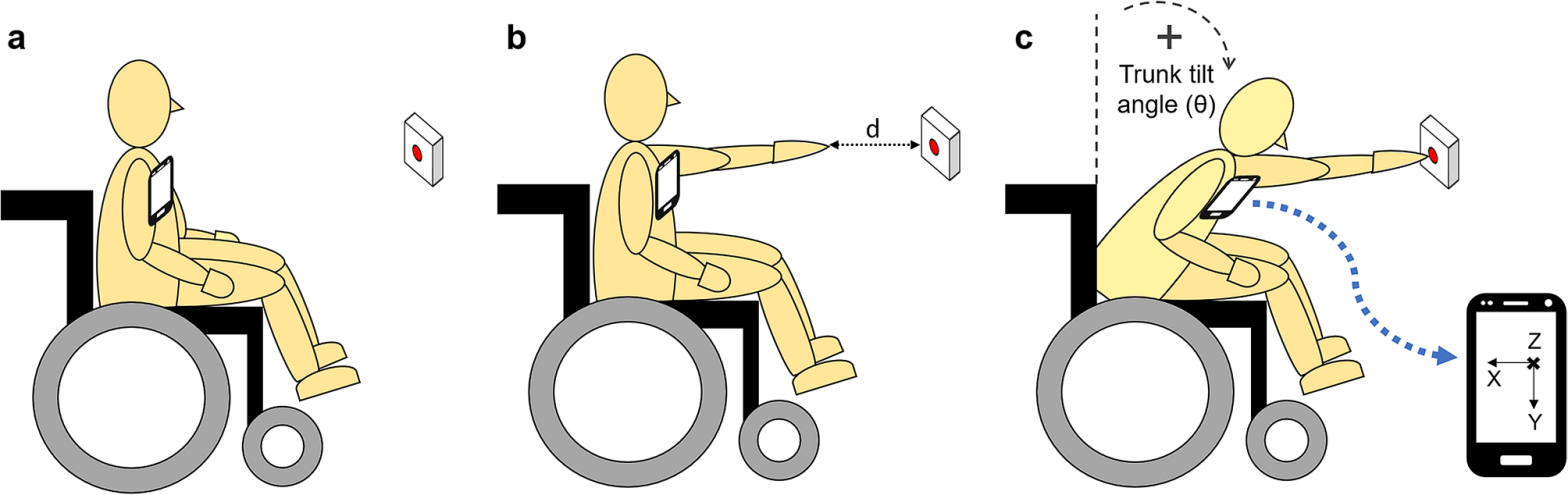
Schematic representation of the subject’s task. The subject is sitting in a wheelchair, and the initial position is at rest, with the arms on the chair’s armrests (**a**), at a distance ‘d’ (15 cm or 30 cm) from the target switch button (**b**). Following a go-signal, the subject raises the left arm and bends the trunk forward to press the button (**c**). A smartphone is attached to the subject’s thorax to collect accelerometer data. The X, Y, and Z directions of the accelerometer are shown.

During the experiments, subjects were sitting in a wheelchair facing a wall. The initial position was at rest, with the back straight and the arms relaxed and resting on the chair’s armrests. The subjects’ task consisted of raising the left arm and, by tilting the trunk forward, touch a 2 cm diameter red circle in the centre of a switch button attached to the wall and immediately return to their initial position (Fig. 1). They were requested to do that as fast as possible in response to an imperative signal (IS), which was a low intensity electrical stimulus (3.6 mA, 0.2 ms duration) applied to the subject’s right little finger. The IS was delivered a few seconds (1–5 s) after a verbal forewarning to be ready. The chair was positioned and, then, fixed, in such a way that the switch was centred on the subject’s midline and the distance between the switch and the subject’s index fingertip when the left arm was fully extended horizontally was either 15 cm or 30 cm.

Each participant repeated the same manoeuvre 20 times (20 trials) at each distance (15 and 30 cm). In five randomly selected trials, a startling auditory stimulus (SAS) was presented at the same time as the IS. The SAS was produced by the discharge of a magnetic coil on top of a metallic platform at maximum stimulator output^41^ (sound intensity of 125 dB, 250 ms duration). Therefore, we had two different conditions: startling auditory stimulus (‘SAS’, 5 trials per subject), and baseline movement (‘non-SAS’, 15 trials per subject). Subjects had already been warned that some trials could be accompanied by an additional stimulus that they should disregard, but they were naïve to the hypothesis being tested.

### Data acquisition

We recorded the EMG activity of eight shoulder, neck, and trunk muscles on the left side of the body: sternocleidomastoid (SCM), middle deltoid (DEL), trapezius (TRA), pectoralis major (PECT), upper abdominal Th6 (ABD), and paraspinal muscles at cervical C3 (PC), thoracic T6 (PT), and lumbar L2 (PL) levels. Disposable adhesive surface electrodes (outer diameter 20 mm; Technomed) were attached over the muscle belly, and the EMG signals were collected using a ten-channel EMG system (Synergy, VIASYS Healthcare UK Ltd., 2005) at a sampling rate of 10 kHz. The ground electrode was placed on the left forearm. In addition, two extra channels were acquired synchronously with the same equipment: the EMG of the left orbicularis oculi muscle (OOc), to detect the blink reflex, and the artifact coming from the wired switch button. This signal was used to calculate the time to complete the task, i.e., the time from the IS to the button press, which we will refer to as the response time. Recording epochs had a duration of 3 s, starting 600 ms before the IS to evaluate relaxation of all muscles before the experiment.

In addition to the EMG recordings, trunk acceleration was measured by the triaxial accelerometer of a smartphone (Samsung Galaxy S5), which was attached to the subjects’ thorax with an elastic band, in the centre of the chest over the sternum, at thoracic 3–4 level (Fig. 1). The accelerometer x-axis was in the medial–lateral direction, the y-axis in the superior-inferior, and the z-axis in the anteroposterior direction. Accelerometer data were sampled at 200 Hz and stored on the phone’s internal memory. Data from smartphone in one participant were lost due to technical problems, so this subject had to be excluded from accelerometry analysis.

### Data processing and analysis

We used the activity of the SCM as a startle indicator, as it has been reported that the presence of short latency SCM activation is a more robust indicator of the effectiveness of SAS activation of the reticulospinal system than the OOc^42^. For this reason, we separated the SAS trials into those where the SCM was activated within 120 ms of the SAS (SCM_≤120 ms_), or later than 120 ms (SCM_>120 ms_), as suggested in previous works^42,43^. We compared the onset latencies of all muscles in non-SAS, SCM_≤120 ms,_ and SCM_>120 ms_ trials, to check if this criterion applied to our task (see Statistical Analysis section below).

Data were processed and analysed using custom software in Matlab (r2018a, Mathworks Inc.) and R (version 3.6.2; http://www.r-project.org).

#### EMG

EMG recordings were band-pass filtered between 40 and 500 Hz to remove movement artifacts, and a notch filter was applied to remove power line interference (50 Hz and harmonics). EMG recordings contaminated by artifacts were manually discarded.

We calculated the EMG onset latencies as the time from the IS to the onset of EMG activity in each muscle. For this purpose, we used an algorithm developed by our group for automatically detecting the EMG onsets^44^, which is based on the ratios of consecutive peaks of the Teager–Kaiser Energy Operator (TKEO)^45,46^. All onsets were visually inspected and manually adjusted, if necessary, to correct for any inaccuracies.

To obtain the EMG envelopes, the signals were full wave rectified, and lowpass filtered at 20 Hz. Then, in addition to the onset latencies, we calculated the following features across each trial and muscle: peak amplitude and area under the curve of the EMG envelope, used as EMG amplitude estimators; and centre of gravity (CoG), defined as the time from the IS to the point where the cumulative energy curve of the EMG envelope reaches the 50% of the total energy, used as another estimator of the temporal distribution pattern of each muscle. Therefore, we had a total of 32 EMG features (4 features × 8 muscles) for each trial in each subject.

#### Accelerometry

Triaxial accelerometry was used to estimate the trunk tilt angle (angle in the sagittal plane) and lateral angle (angle in the coronal plane). As the movement was mainly rotational, we assumed that linear acceleration was negligible, so the tilt (θ) and lateral (φ) angles at each time point, with respect to the vertical axis of the body, could be derived as follows:

1 $$\theta = \cos^{ - 1} \left( {\frac{{a_{z} }}{{\sqrt {a_{y}^{2} + a_{z}^{2} } }}} \right) - \frac{\pi }{2},$$

2 $$\varphi = \frac{\pi }{2} - \cos^{ - 1} \left( {\frac{{a_{x} }}{{\sqrt {a_{x}^{2} + a_{y}^{2} } }}} \right),$$

where a_x_, a_y_, and a_z_ are the values of the x, y, and z axes of the accelerometer, respectively, at each time point, and the angles, θ and φ, are expressed in radians. Defined in this way, θ > 0 corresponds to forward tilt, and θ < 0 to backward tilt, while φ > 0 represents a lateral deviation to the right, and φ < 0 to the left.

**Figure 2:**
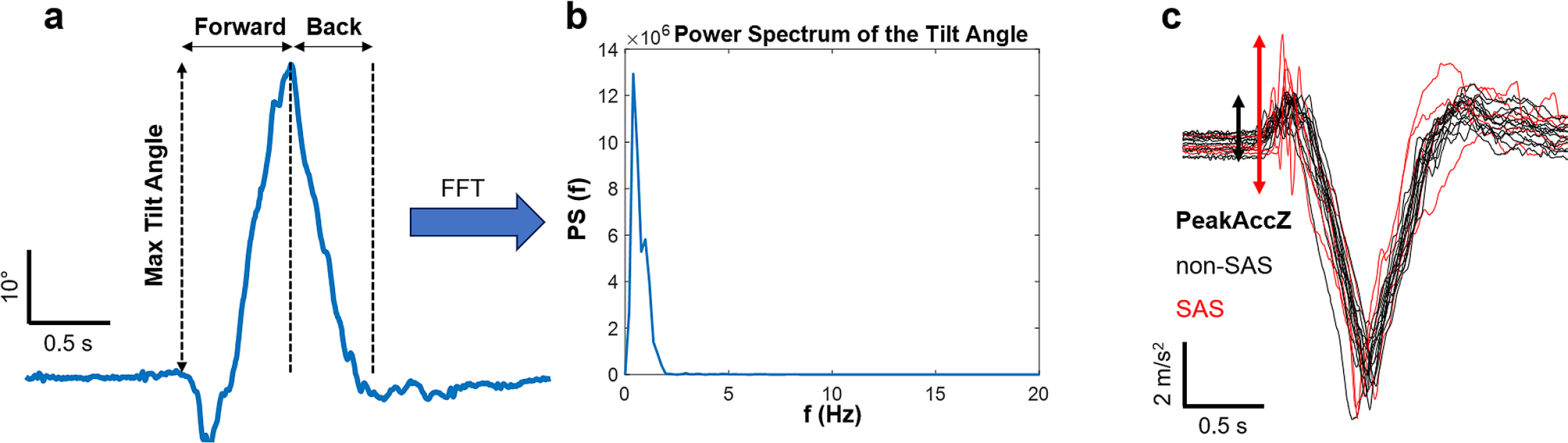
Example of the raw trunk tilt angle signal, *θ*, derived from the triaxial accelerometry of the smartphone (**a**), the power spectrum of this angle signal (**b**), and the z-axis accelerometer data showing the peak at the beginning of the movement (*PeakAccZ*) for non-SAS trials (black) and SAS trials (red) (**c**).

Next, we extracted different features from the angle signals of each trial. First, the angle signals were lowpass filtered below 2 Hz, to reduce high-frequency noise. The maximum absolute value of the lateral angle was taken as a measure of the trunk lateral deviation from the central sacral line. From the tilt angle signal, we calculated the maximum tilt angle (i.e., trunk forward inclination angle), the total movement duration, and the durations and angular velocities of the forward movement (from the beginning of the movement to the time of the maximum tilt angle) and the backward movement (from the time of the maximum tilt angle to movement end) (Fig. 2a). We also computed the power spectrum (PS) of the raw tilt angle signal of each trial as the squared magnitude of the Fast Fourier Transform (FFT) (Fig. 2b). This was used to extract spectral features such as the median frequency, third quartile frequency (fQ3), 95th percentile frequency (fmax), and the ratio between the energies in the frequency bands > 3 Hz and ≤ 3 Hz (RATIOHL).

Moreover, as we were interested in the initiation of the movement, we measured the maximum peak-to-peak distance in the first 200 ms of the signals from the three accelerometer axes, as illustrated in Fig. 2c. We will refer to these features as PeakAccX, PeakAccY, and PeakAccZ.

### Statistical analysis

Data were averaged across trials to obtain a single measure for each variable and subject at each distance (15 cm vs 30 cm) and condition (non-SAS vs SAS). We then calculated the group mean and standard deviation (SD) to report descriptive statistics of each feature across distance and condition. Results are reported as Mean (SD).

First, we investigated whether the early activation of SCM was a reliable indicator of the startle reflex in our task, in which the SCM is also involved in the neck movement that accompanies trunk flexion. To do that, multiple pairwise Wilcoxon tests were run to compare the onset latencies of all muscles in non-SAS, SCM_≤120 ms,_ and SCM_>120 ms_ trials at each distance, and thus decide whether it was appropriate to disregard SCM_>120 ms_ trials, depending on whether they also induced a latency shortening. After that, only two conditions (non-SAS vs SAS) were considered, since there were too few subjects with SCM_>120 ms_ trials to maintain the three categories in the main analysis described below.

A two-way repeated measures ANOVA was applied to each variable, to investigate the effects of distance, condition, and the interaction between distance and condition. Q-Q plots were used to determine if the residuals were approximately normally distributed. The level of significance in all the statistical tests was set to 0.01 in order to restrict the number of false positives, since we are dealing with a large number of parameters (47 parameters in total: 1 the response time from the switch button, 14 from the accelerometer, and 32 from the EMG) at 2 distances (15 cm/30 cm) and 2 conditions (non-SAS/SAS).

For the variables showing statistically significant differences between non-SAS and SAS condition, we calculated the percentage change for each subject, and then the mean and standard deviation of percentage change. We also calculated the percentage of subjects presenting at least a 5% and a 10% difference in that feature, to evaluate whether the differences in the average values were consistent and repeatable across subjects.

The correlation of the startle changes in each of the variables with age and gender were analysed with Spearman’s rank correlation coefficient and Mann–Whitney U-test, respectively.

## Results

### Characteristics of EMG activity and movement description

**Figure 3:**
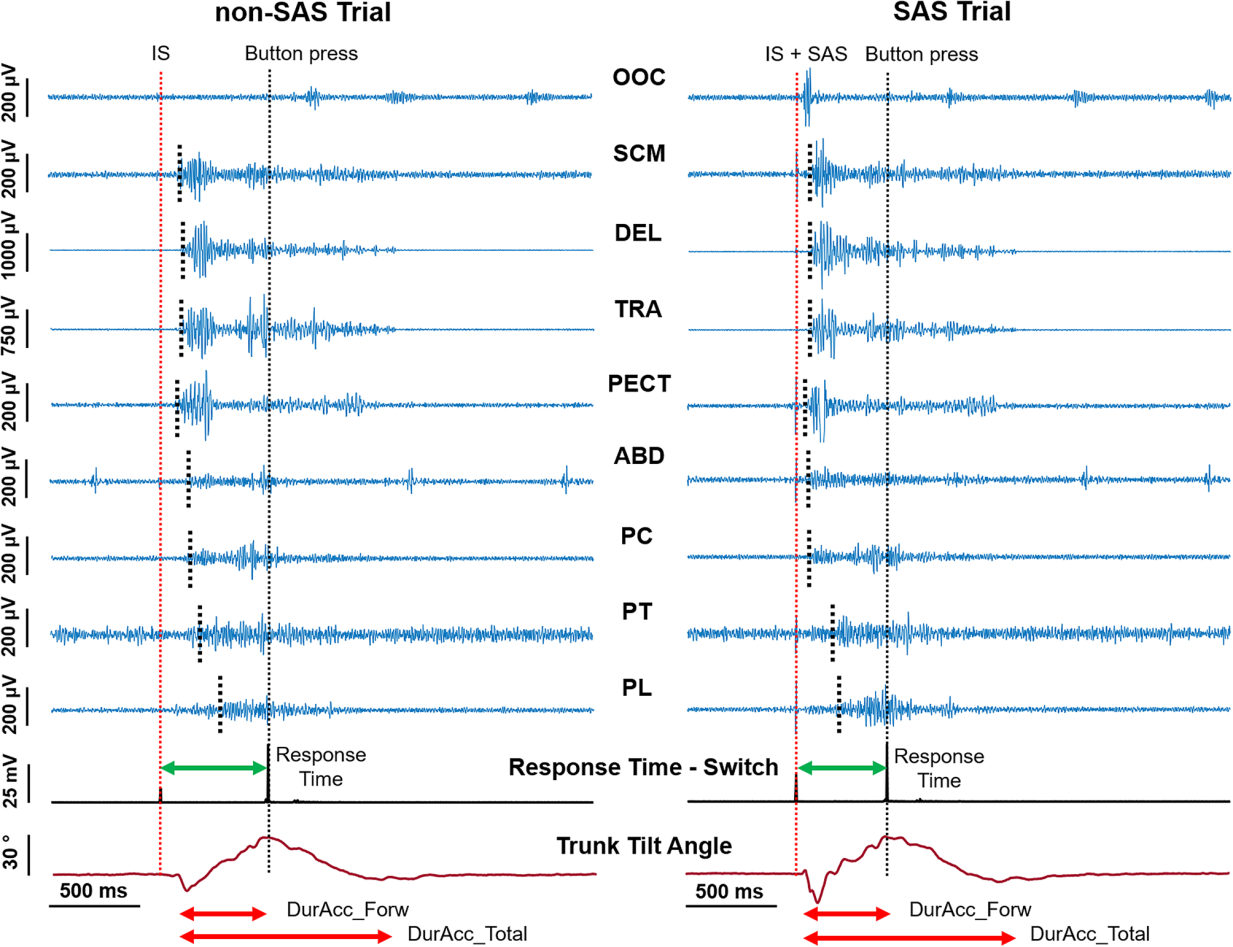
Example of the signals recorded during the trunk movement task at 15 cm. From top to bottom: EMG recordings of the OOc, SCM, DEL, TRA, PECT, ABD, PC, PT, and PL; artifact signal from the switch button, indicating the response time, and trunk tilt angle (θ) derived from the smartphone accelerometer. Small black dotted lines indicate the onset latencies of each muscle. The vertical dotted lines indicate the imperative signal (IS) (red), and the button press time (black). The response time until the button press is indicated with green arrows. The red arrows indicate the duration of the forward movement (DurAcc_Forw) and the total duration of the movement (DurAcc_Total) measured from the accelerometer data.

All subjects performed the task without difficulty. Because of artifact contamination, data of ABD from one participant and of PC from another participant were excluded from the analysis. Examples of the signals recorded in a non-SAS and a SAS trial are shown in Fig. 3. The trunk tilt angle (θ) indicates the movement trajectory: from a resting position, the subjects first moved slightly backward, as indicated by angle values below the baseline, and then they flexed the trunk forward until reaching the maximum tilt angle when pressing the switch button. Finally, they moved backward to the original position.

The muscle recruitment pattern looked qualitatively similar in non-SAS and SAS trials: the neck and shoulder muscles (SCM, DEL, TRA, and PECT) were activated first and reached their maximum activity in the early phase of the movement. The ABD showed a prolonged low activity burst. The PC, PT, and PL muscles were activated sequentially in that order. The SAS elicited a blink reflex in 90% of the SAS trials in the dataset, evidenced in the EMG of the OOc as a short high amplitude burst within the first 45 (10) ms.

**Figure 4:**
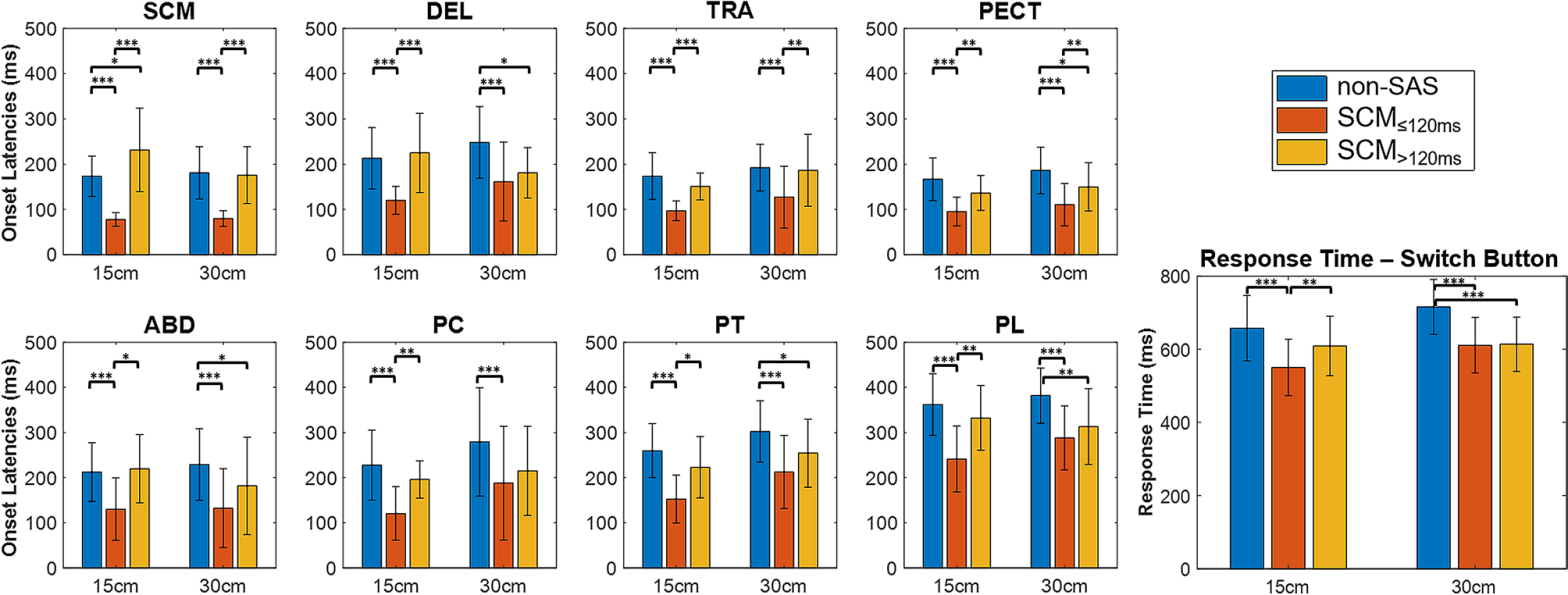
Barplots showing the response time (switch button) and onset latencies of the 8 muscles, in non-SAS trials (blue), and SAS trials with or without an early response in SCM (red: SCM_≤120 ms_, yellow: SCM_>120 ms_). Mean and standard deviation of the total values in the dataset are shown. Statistical significance levels for pairwise comparisons using Wilcoxon tests are shown: 0.05*, 0.01*, 0.001***. Only the SCM_≤120 ms_ trials show significant reductions in all the muscle onset latencies and the response time.

As exemplified in Fig. 3, the SAS reduced the response time, as well as all the muscle onset latencies. However, this only happened when an early response (≤ 120 ms) was observed in the SCM, i.e., in SCM_≤120 ms_ trials, as shown in Fig. 4. For this reason, the SCM response proved to be an effective startle indicator and, from this point on, the SCM_>120 ms_ trials were discarded since consistent startle responses were not elicited in these trials. Therefore, all the results for the SAS condition include only SCM_≤120 ms_ trials. This SCM_≤120 ms_ response was present in 78% (129 trials) of the SAS trials in the dataset. Out of the 33 participants, 23 showed a SCM_≤120 ms_ response in at least 80% of the trials, while 5 only showed a SCM_≤120 ms_ response in less than half of the trials (including both distances). Data from participants with less than two SCM_≤120 ms_ trials at each distance were excluded from further analysis: for the distance of 15 cm, four subjects were discarded, while, at 30 cm, only one subject was discarded.

Table 1 shows a summary of all data and the results of comparative statistical tests (two-way repeated measures ANOVA) for differences related to condition (SAS vs non-SAS) and target distance (30 cm vs 15 cm). Table 2 shows the mean percentage change in response time and each EMG and accelerometer feature showing statistically significant differences (p < 0.01) between non-SAS and SAS trials, as well as the percentage of subjects showing differences > 5% or > 10% in SAS with respect to non-SAS values.

**Table 1:** Outcome measures in non-SAS and SAS trials, both at 15 cm and 30 cm. The first four columns show the Mean (SD) for each condition, and the last four columns show the results of the statistical analysis, using two-way repeated measures ANOVA with Startle effect and Distance as factors. p-values < 0.01 are highlighted in bold.

Parameter	Mean (SD) 15 cm		Mean (SD) 30 cm		p-values		F-statistic	
	non-SAS	SAS	non-SAS	SAS	Startle	Distance	Startle	Distance
**Switch button**								
Response time (ms)	650 (85)	554 (75)	721 (74)	615 (73)	** < 0.001**	** < 0.001**	136.37	42.45
**EMG—onset latencies (ms)**								
SCM	168 (43)	77 (15)	180 (59)	79 (17)	** < 0.001**	0.291	239.61	1.13
DEL	202 (59)	119 (31)	246 (81)	162 (89)	** < 0.001**	** < 0.001**	105.2	17.63
TRA	165 (47)	96 (22)	191 (51)	123 (66)	** < 0.001**	**0.002**	67.04	9.85
PECT	161 (45)	95 (32)	187 (53)	111 (47)	** < 0.001**	**0.005**	128.28	8.44
ABD	202 (60)	128 (69)	229 (82)	131 (89)	** < 0.001**	0.584	74.85	0.3
PC	223 (79)	121 (60)	278 (121)	187 (128)	** < 0.001**	** < 0.001**	68.37	17.7
PT	251 (56)	150 (52)	300 (66)	208 (78)	** < 0.001**	** < 0.001**	115.01	31.83
PL	350 (63)	246 (70)	379 (60)	285 (70)	** < 0.001**	**0.003**	123.95	9.31
**EMG—CoG (ms)**								
SCM	668 (143)	570 (167)	691 (95)	613 (132)	** < 0.001**	0.19	38.74	1.75
DEL	576 (110)	483 (95)	640 (89)	543 (88)	** < 0.001**	** < 0.001**	101.96	27.64
TRA	714 (123)	619 (137)	721 (99)	627 (106)	** < 0.001**	0.901	90.73	0.02
PECT	609 (152)	502 (150)	655 (135)	537 (132)	** < 0.001**	0.126	81.98	2.38
ABD	726 (139)	607 (147)	757 (150)	657 (170)	** < 0.001**	0.147	52.17	2.15
PC	682 (122)	587 (143)	745 (150)	638 (133)	** < 0.001**	**0.003**	79.05	9.5
PT	730 (97)	632 (88)	790 (97)	675 (105)	** < 0.001**	**0.001**	122.03	11.46
PL	694 (95)	599 (106)	756 (92)	663 (90)	** < 0.001**	** < 0.001**	51.57	17.09
**EMG—areas (μV s)**								
SCM	30 (17)	36 (21)	39 (21)	45 (24)	** < 0.001**	** < 0.001**	21.12	45.98
DEL	242 (118)	264 (139)	341 (164)	348 (171)	0.097	** < 0.001**	2.82	106.02
TRA	127 (83)	138 (89)	169 (113)	174 (114)	0.152	** < 0.001**	2.08	70.92
PECT	58 (42)	67 (46)	79 (58)	84 (64)	**0.005**	** < 0.001**	8.32	18.63
ABD	21 (17)	25 (17)	30 (27)	34 (28)	0.013	** < 0.001**	6.39	18.26
PC	28 (28)	34 (39)	41 (34)	46 (39)	**0.001**	** < 0.001**	10.91	35.08
PT	40 (24)	46 (32)	52 (25)	53 (28)	0.063	** < 0.001**	3.56	32.91
PL	20 (8.6)	20.4 (9.2)	29 (10)	30 (10)	0.652	** < 0.001**	0.21	93.59
**EMG—peak amplitudes (μV)**								
SCM	102 (63)	133 (78)	133 (88)	155 (81)	** < 0.001**	** < 0.001**	22.49	26.92
DEL	834 (512)	967 (524)	1114 (578)	1170 (583)	** < 0.001**	** < 0.001**	12.81	82.86
TRA	402 (245)	485 (284)	499 (346)	564 (375)	** < 0.001**	** < 0.001**	15.44	37.51
PECT	212 (137)	321 (275)	256 (142)	321 (208)	** < 0.001**	0.508	18.68	0.44
ABD	88 (120)	99 (92)	115 (171)	138 (170)	0.22	0.045	1.53	4.16
PC	100 (105)	119 (112)	145 (129)	161 (138)	0.051	** < 0.001**	3.93	15.66
PT	141 (108)	163 (113)	170 (104)	190 (119)	** < 0.001**	** < 0.001**	11.74	28.99
PL	87 (42)	88 (38)	118 (59)	120 (50)	0.83	** < 0.001**	0.05	49.44
**Accelerometry**								
Duration (s)	1.17 (0.16)	1.16 (0.18)	1.31 (0.15)	1.28 (0.14)	0.41	** < 0.001**	0.69	37.12
Duration forward (s)	0.53 (0.05)	0.53 (0.06)	0.58 (0.05)	0.58 (0.06)	0.466	** < 0.001**	0.54	38.98
Duration backward (s)	0.63 (0.12)	0.63 (0.16)	0.72 (0.12)	0.71 (0.12)	0.507	** < 0.001**	0.44	20.73
Max. trunk tilt angle (°)	27.3 (5)	27.9 (6.2)	39.2 (6.6)	41 (6.8)	0.039	** < 0.001**	4.4	420.39
Max. lateral deviation (°)	3 (1.2)	3.1 (1.4)	3.4 (1.4)	3.4 (1.4)	0.638	0.023	0.22	5.38
Forward velocity (°/s)	52 (11)	54 (14)	69 (16)	72 (16)	0.029	** < 0.001**	4.9	170.2
Backward velocity (°/s)	46 (11)	48 (15)	57 (15)	61 (18)	0.075	** < 0.001**	3.26	64.83
PeakAccX (m/s^2^)	2.7 (1.7)	4.1 (2.9)	2.7 (2)	3.6 (2.8)	** < 0.001**	0.461	41.3	0.55
PeakAccY (m/s^2^)	2.2 (1.5)	4.3 (3.6)	2.6 (1.9)	4.6 (3.4)	** < 0.001**	0.057	76.94	3.72
PeakAccZ (m/s^2^)	2.2 (1.2)	4.2 (2.9)	2.6 (1.6)	4.5 (3)	** < 0.001**	0.034	72.99	4.64
Median frequency (Hz)	1.13 (0.2)	1.23 (0.38)	0.96 (0.11)	0.99 (0.14)	0.028	** < 0.001**	5.03	38.29
fQ3 (Hz)	1.59 (0.67)	2.3 (2.3)	1.28 (0.37)	1.49 (0.68)	**0.01**	**0.003**	6.94	9.62
fmax (Hz)	5.4 (2.6)	8.1 (4.8)	4.3 (2.9)	5.9 (4.1)	** < 0.001**	** < 0.001**	44.16	18.08
RATIOHL (%)	12.7 (8.8)	21 (20)	8.7 (5.6)	13 (11)	** < 0.001**	** < 0.001**	18.56	13.96

**Table 2:** Mean percentage changes with respect to non-SAS values, and percentage of subjects showing at least 5% and 10% differences, for all the features showing statistically significant differences (p < 0.01) between non-SAS and SAS trials, at 15 cm and 30 cm.

Parameter	p-value Startle factor ANOVA	Differences: Higher ( >) or Lower ( >) in SAS trials	Mean (SD) Percentage Change		% Subjects showing differences > 5%		% Subjects showing differences > 10%	
			15 cm	30 cm	15 cm	30 cm	15 cm	30 cm
**Switch button**								
Response time (ms)	< 0.001	<	15 (7)	15 (8)	97	78	77	72
**EMG—onset latencies (ms)**								
SCM	< 0.001	<	52 (14)	52 (16)	100	100	100	100
DEL	< 0.001	<	37 (19)	35 (20)	97	91	90	88
TRA	< 0.001	<	38 (20)	31 (48)	93	88	93	88
PECT	< 0.001	<	39 (16)	40 (19)	97	94	97	94
ABD	< 0.001	<	36 (31)	42 (26)	93	90	89	90
PC	< 0.001	<	44 (21)	36 (26)	93	94	93	91
PT	< 0.001	<	39 (20)	30 (24)	97	88	93	81
PL	< 0.001	<	30 (16)	25 (15)	90	91	90	84
**EMG—CoG (ms)**								
SCM	< 0.001	<	15 (10)	12 (13)	90	78	67	59
DEL	< 0.001	<	16 (7)	15 (9)	93	84	87	75
TRA	< 0.001	<	14 (9)	13 (8)	87	91	63	75
PECT	< 0.001	<	18 (10)	18 (12)	87	91	80	81
ABD	< 0.001	<	17 (12)	14 (13)	79	84	79	74
PC	< 0.001	<	14 (10)	14 (6)	83	97	77	72
PT	< 0.001	<	13 (6)	14 (10)	87	88	73	72
PL	< 0.001	<	14 (8)	12 (10)	97	88	70	56
**EMG—areas (μV s)**								
SCM	< 0.001	>	20 (23)	18 (24)	67	75	63	59
PECT	0.005	>	15 (21)	8 (22)	57	56	40	41
PC	0.001	>	20 (26)	12 (15)	70	63	57	47
**EMG—peak amplitudes (μV)**								
SCM	< 0.001	>	34 (32)	26 (42)	90	69	83	63
DEL	< 0.001	>	19 (21)	7 (15)	77	44	63	28
TRA	< 0.001	>	25 (27)	15 (15)	83	78	73	56
PECT	< 0.001	>	44 (42)	27 (45)	90	75	90	59
PT	< 0.001	>	18 (23)	14 (22)	77	66	63	59
**Accelerometry**								
PeakAccX (m/s^2^)	< 0.001	>	58 (74)	37 (48)	86	74	83	74
PeakAccY (m/s^2^)	< 0.001	>	95 (79)	83 (65)	93	97	93	97
PeakAccZ (m/s^2^)	< 0.001	>	89 (64)	73 (63)	97	94	97	81
fQ3 (Hz)	0.01	>	31 (63)	15 (26)	59	48	45	39
fmax (Hz)	< 0.001	>	47 (40)	41 (71)	90	71	90	65
RATIOHL (%)	< 0.001	>	55 (67)	46 (72)	83	71	79	68

### Effect of target distance

The response time was longer at 30 cm than at 15 cm, as the target was further away. Muscle latencies and CoG, measured in milliseconds from the IS, tended to be longer as well, but significant changes were found only for the DEL and the three paraspinal muscles in both parameters, and the TRA and PECT for onset latencies alone (all p ≤ 0.005). On the other hand, the EMG amplitude estimators showed larger values at 30 cm compared to 15 cm, with statistically significant differences at a level of 0.01 in all muscles (p ≤ 5 × 10^–5^) except for the peak amplitude of PECT (p = 0.508) and ABD (p = 0.045).

Data from the smartphone accelerometer showed that all subjects performed straightforward movements, with minimal lateral deviation, always < 5°. The trunk tilt angles reached during the movement were higher at 30 cm than at 15 cm, with mean values of 27 (5)° in non-SAS trials and 28 (6)° in SAS trials at 15 cm, in contrast to 39 (7)º in non-SAS trials and 41 (7)° in SAS trials at 30 cm. The total movement duration, as well as the durations of both forward and backward phases, increased with the distance (p ≤ 3 × 10^–8^), as the subjects were further away from the target. Subjects were faster on trials at 30 cm, as denoted by the increase in angular velocities (p ≤ 4 × 10^–12^). The size of the peak in the first 200 ms for each of the three accelerometer axes (PeakAccX, PeakAccY, and PeakAccZ) did not change with target distance (p ≥ 0.03). The frequency content of the accelerometer signal was lower in the movements at 30 cm than at 15 cm, according to all the extracted spectral parameters (p ≤ 0.003).

### Effect of the startling stimulus

**Figure 5:**
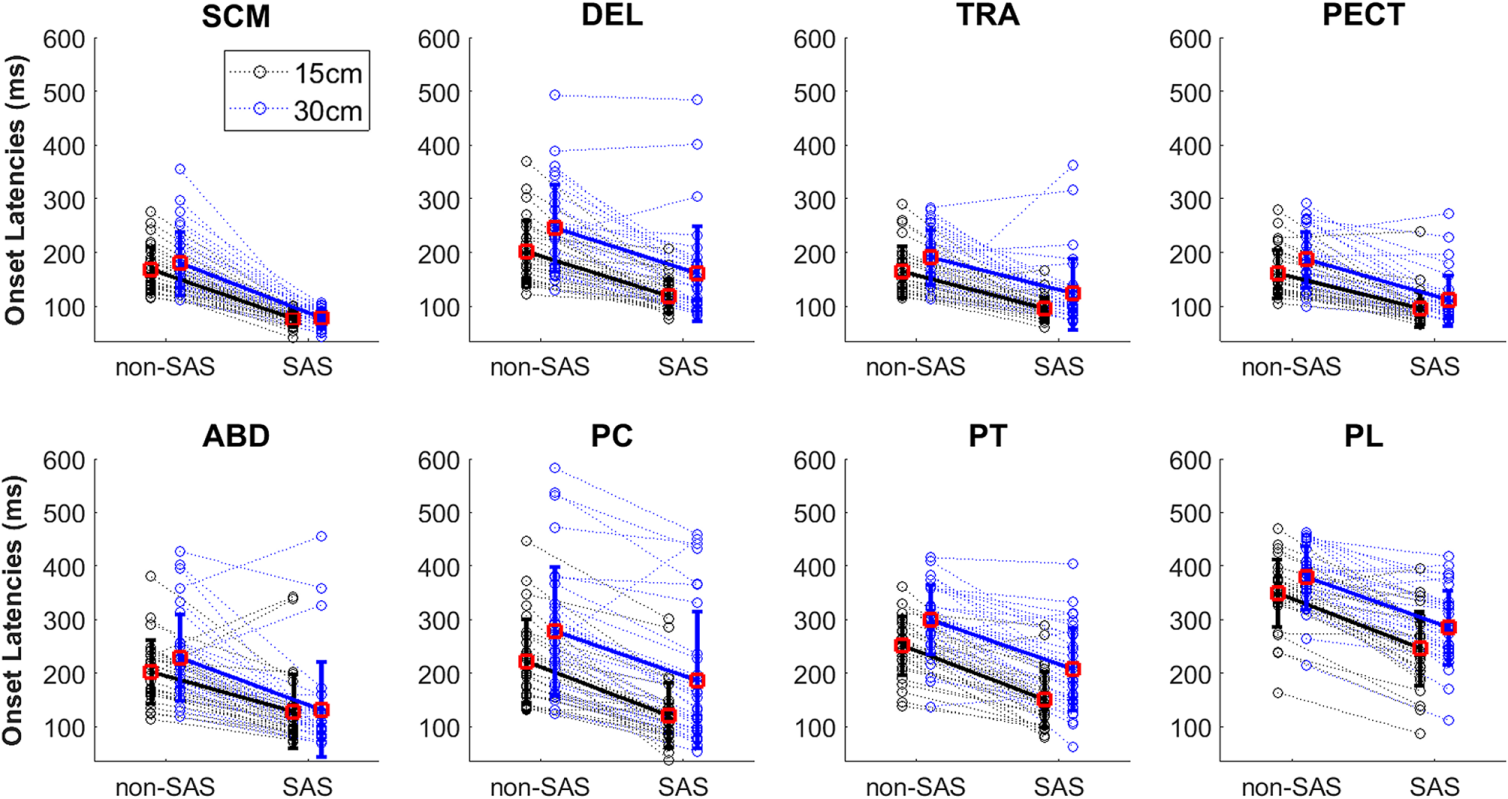
Onset latencies for the eight muscles and all the subjects, during the trunk movement task in non-SAS and SAS trials, at 15 cm (black) and 30 cm (blue). Each dotted line represents one subject, while the mean values are shown in wider lines, with error bars corresponding to the standard deviation.

The response time, i.e., time of button press, was significantly reduced in SAS vs non-SAS trials: 554 (75) ms vs. 650 (85) ms at 15 cm, and 615 (73) vs. 721 (74) ms at 30 cm (p < 10^–16^). The mean percentage reduction was 15% (Table 2), ranging from 2 to 35%. EMG onset latencies were also significantly shorter in SAS than in non-SAS trials (p ≤ 2 × 10^–12^ for all muscles), with mean percentage changes between 25 and 52% (Table 2). This noticeable latency shortening was consistent and repeatable between subjects: more than 88% of the participants had differences > 5%, and more than 81% had differences > 10% for all muscles. This is illustrated in Fig. 5, which shows the onset latencies in SAS and non-SAS conditions for all subjects and muscles, both at 15 cm and 30 cm. Note that, as shown in Fig. 5, the pattern of muscle recruitment does not seem modified with distance or condition: the SCM is the first muscle to be activated, followed by the PECT and TRA, then the DEL and ABD, and, finally, the sequential activation of the PC, PT, and PL. However, this was not quantified and was therefore not confirmed statistically. The CoG were also significantly reduced for all muscles (p ≤ 2 × 10^–8^), but the magnitudes of the differences were lower than those of EMG onset latencies, with mean percentage reductions between 12 and 18% (Table 2). The onset latencies indicate when each muscle is activated, whereas the CoG indicate when they reach the 50% of their total energy, and thus give an idea of how the activity of this muscle is distributed along the movement. In all distances and conditions, the earlier CoG occurs in shoulder muscles, DEL and PECT. These are followed by two neck muscles, SCM and PC, and finally the scapular and trunk stabilizers, TRA, ABD, PL, and PT (Table 1).

The mean values of the EMG amplitude estimators were higher in SAS than in non-SAS trials for all muscles, but differences were only significant for SCM and PECT for both the area and peak amplitude, in addition to the area of PC, and the peak amplitude of DEL, TRA, and PT (all p ≤ 0.005). The increase in EMG amplitude estimators was found in a smaller proportion of subjects (Table 2), showing much less consistency than the reduction of latencies. The peak amplitudes of SCM and PECT had the highest mean percentage changes (≥ 26%), and they were present in the highest number of subjects (between 59 and 90% of the subjects had differences > 10%).

**Figure 6:**
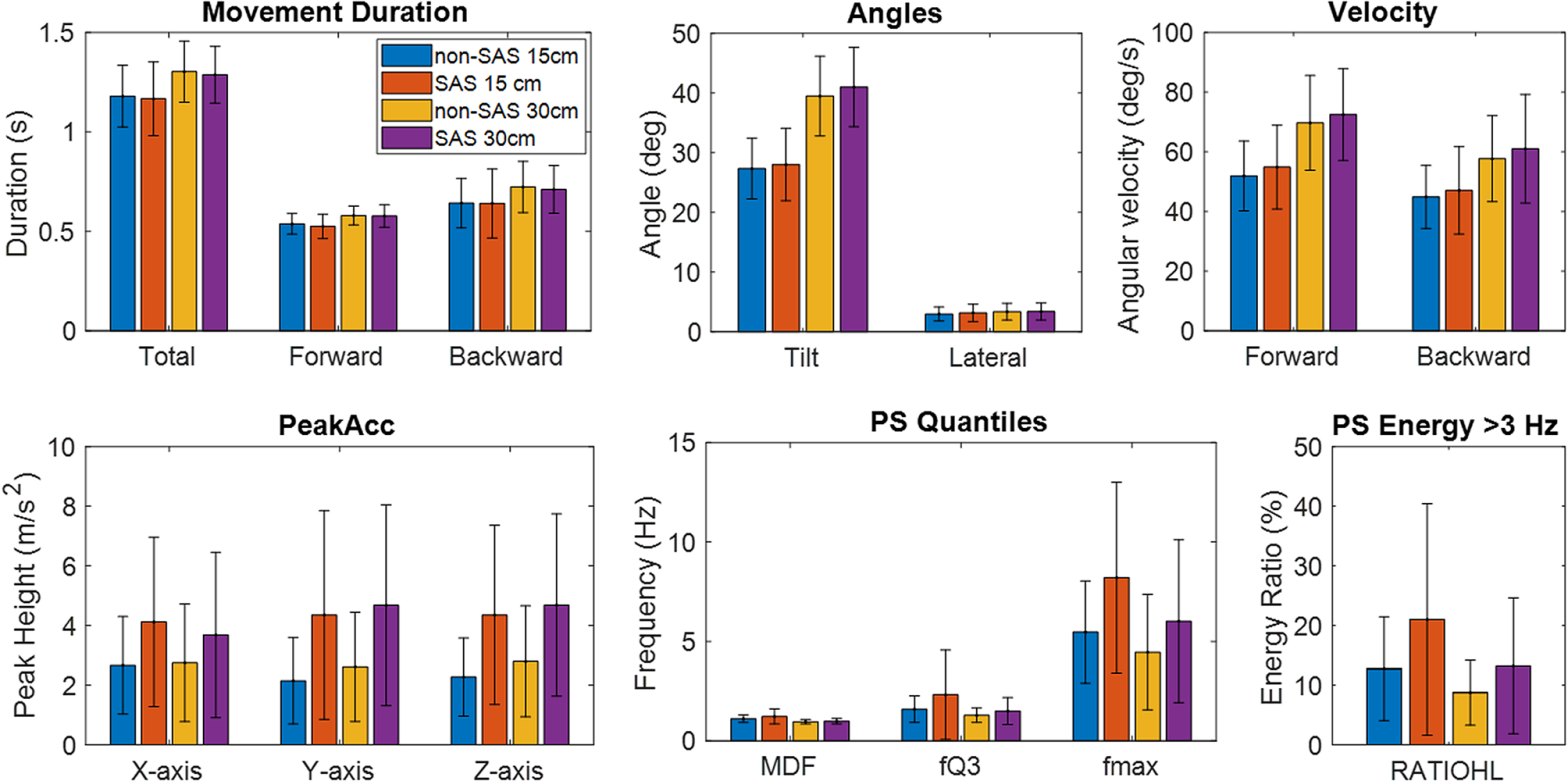
Barplots showing the mean and standard deviation of all the parameters extracted from the smartphone accelerometer, for the two distances (15 cm vs 30 cm) and conditions (SAS vs non-SAS).

Data extracted from the smartphone accelerometer (Fig. 6) revealed no significant differences between non-SAS and SAS trials for movement durations (p > 0.4), tilt angle (p = 0.04), lateral angle (p = 0.64), and angular velocities (p ≥ 0.03) (Table 1). Therefore, although the onset latencies and the response time were reduced in SAS trials, the movement duration, as indicated by the accelerometer traces, did not change (Fig. 3).

PeakAccX, PeakAccY, and PeakAccZ were significantly larger in SAS than in non-SAS trials in the three accelerometer axes (p ≤ 7 × 10^–9^). Such increase was very consistent across subjects, especially in Y and Z-axes, with mean percentage changes ≥ 73% and differences > 10% at least in 81% of the subjects. The spectral parameters also increased in SAS trials, indicating a higher frequency content of the tilt angle signal derived from the accelerometer data when the SAS was presented (Fig. 6). These differences were especially significant in the 95th percentile frequency (fmax*)* and the energy ratio (RATIOHL) (p ≤ 7 × 10^–5^), but also for the 3rd quartile frequency (fQ3) (p = 0.01), while they were not significant at a level of 0.01 for the median frequency (p = 0.03) (Table 1). The increases in fmax and RATIOHL were consistent among subjects (mean percentage changes ≥ 41%, and differences > 10% in 65–90% of the subjects), unlike what happened in fQ3, where differences > 10% occurred only in 39–45% of the subjects (Table 2).

### Interaction between distance and condition

No interaction effects were found between the two factors entered in the analysis (Condition: SAS/non-SAS and Distance: 15 cm/30 cm) for any of the EMG or the accelerometer features, with p-values ranging from 0.09 to 0.996.

### Correlation with age and gender

At a level of significance of 0.01, no correlations were found between the changes in any of the variables and age or gender (p > 0.02 for all parameters). From the 94 correlation tests (47 parameters at 2 distances), only two p-values were < 0.05 (but ≥ 0.04) for gender correlation, and four p-values were < 0.05 (but ≥ 0.02) for age correlation, which are less than would be expected purely by chance.

## Discussion

The StartReact phenomenon has long been investigated in different movements^31–39^, and it is well-known that a prepared response can be triggered at short latency when a SAS is presented^47^. Here we show that this phenomenon also occurs in postural muscles involved in trunk flexion in reaching tasks while sitting. We examined eight shoulder, neck, and trunk muscles: the SCM, responsible for neck flexion; the DEL and PECT, prime movers for shoulder flexion; the TRA, the main contributor to scapular stability and mobility; and the ABD, PC, PT, and PL, muscles involved in bending the trunk forward and maintaining the upright posture. In SAS trials, we found significant reductions in response time and onset latencies of the eight muscles under study, with similar percentage changes (around 30–40%, Table 2) in most of them. Therefore, in this task, the SAS induces a similar effect in prime movers and postural muscles, suggesting that all of them are modulated by the neural pathway activated in the StartReact effect. Although there is still debate about the mechanisms of this effect^47^, a plausible hypothesis is the subcortical storage explanation, which considers that, in preparation of voluntary actions, sufficient detail of the movement is stored in the brainstem and spinal centres so that the motor program can be triggered even without cortical command^32^. This interpretation was given in a previous study about the startle effect on the sit-to-stand movement^36^, where the authors found that the SAS affected prime movers and postural muscles alike, just as in our study, and reasoned that all the examined muscles had a common modulation at a subcortical level. This would be a possible explanation of the StartReact effect in the present study, although, with the current data, we cannot discard alternative hypothesis such as cortical contributions^48^.

The greatest shortening of onset latency (52%) occurs in the SCM due to startle-related activation, since, as it is well-known, unexpected loud auditory stimuli elicit SCM contraction. For this reason, the early activation of this muscle is considered a reliable indicator of the startle reflex^42,43^. However, unlike in previous studies, the SCM in our experiment was activated not only in response to SAS, but, also, as a postural muscle needed for task execution, as it is the responsible for the flexion of the neck that accompanies trunk flexion. Even so, the SCM activation at short latencies demonstrated to be indicative of the startle response: only when this muscle was activated within 120 ms of the SAS (SCM_≤120 ms_ trials) a significant reduction in the onset latencies of all the muscles was observed (Fig. 4). Although data for the SCM in Fig. 5 show a reduced dispersion because of the selection of only SCM_≤120 ms_ trials, this selection helped to ensure that the startle reflex circuitry had been activated to trigger the prepared response.

We found enhanced EMG activity in SAS trials than in non-SAS trials, although the differences were significant only for some muscles, especially the SCM and PECT, and only occurred in a proportion of subjects. Previous works have reported increased EMG responses following the presentation of a SAS in the SCM and the wrist flexor^41,42^, as well as the tibialis anterior^36^, hypothesizing that the reason might be a sustained increase in subcortical motor pathways excitability^41^. We expected different effects in the neck and arm muscles compared to trunk muscles, as the latter have a postural function to maintain equilibrium, and thus should be less sensitive to perturbations. On the other hand, the EMG amplitudes for all the examined muscles were significantly higher at 30 cm than at 15 cm, indicating that the subjects had to exert a greater muscle force to cover a longer distance.

Here, we have combined EMG and smartphone accelerometer data as a method to simultaneously analyse muscle activity and movement patterns related to trunk flexion in an arm reaching task. EMG, as a neuromuscular assessment technique, has been extensively used in StartReact studies, and kinematic measures or accelerometer signals have also been employed for measuring response time or movement duration in this context^31,40,49^. However, in these cases, the data are usually recorded with uniaxial accelerometers, which limits movement analysis to a few measures, whereas triaxial accelerometers could provide more precise spatial information. Moreover, most of previous kinematic studies of trunk function relied on expensive specialized equipment such as motion capture systems, which limits their application in the clinical setting. In contrast, smartphones are available worldwide, and they are simple cost-effective tools that have accurate sensors and powerful data-processing capabilities, so they would be easy to implement in the clinical practice. This work is a first proof-of-concept of how the smartphone accelerometers could help in the assessment of trunk movements.

The smartphone accelerometer allows recording movement signals, which likely reflect postural control and trunk stability, as suggested by Queralt et al.^36^, since the device is attached to the subjects’ thorax and tracks the trunk displacement. Firstly, the data derived from the smartphone accelerometer allowed us to monitor the trunk tilt angle and the lateral angle at any point during the movement. With that, we calculated the maximum tilt angle reached by each subject at each distance, and the maximum lateral deviation. This lateral deviation was found to be very small in all situations because the participants were able-bodied subjects, so they made accurate, straightforward movements with no difficulties. However, this may not be the case in patients with neurological injuries, as they may need to adopt compensatory strategies to prevent instability when performing the experimental task reported in this study, which may be reflected in greater lateral deviations. Secondly, we conducted a spectral analysis of the tilt angle signal, which showed that higher frequency components appear following the presentation of a SAS. This high frequency results from less smooth movements, and could indicate reduced control in SAS trials, possibly related to an involuntary reaction to the unexpected stimulus or to the faster movement initiation. This change in frequency might also relate to additional drive associated with the startle reflex pathways adding to the voluntary initiation drive, but further research would be needed to investigate this. The signals at 30 cm had slightly lower frequency content than at 15 cm, but the reason could be that, at 30 cm, movements are longer and thus the effect of the transients, which have higher frequency content, is reduced.

We have also found an interesting parameter, the height of the peak that occurs in the accelerometer signal at the beginning of the movement, measured in the three accelerometer axes (PeakAccX, PeakAccY, and PeakAccZ). We think that this feature reflects the anticipatory movement preceding the trunk flexion, which significantly increases in SAS trials (Fig. 2c) because the subjects involuntary jump or wince in response to the unexpected loud sound. Therefore, the size of these peaks in the accelerometer signal could be a quantifiable surrogate of the magnitude of body movement generated by the startling stimulus. On the other hand, the increase in the height of these short duration peaks in SAS trials may contribute to the presence of higher frequency components in the spectral domain, suggesting some kind of destabilization during movement initiation.

The accelerometer signal also enabled the calculation of the total movement duration, and the durations and angular velocities of the forward and backward movements. None of these features changed when a SAS was presented, even though the response time and onset latencies were markedly shortened. This indicates that the time to complete the task is shorter because muscles are activated earlier and thus the movement starts sooner, not because the execution is faster since the duration does not change. This explanation is supported by Fig. 3, which shows that the onset latencies of all muscles and the button press time are closer to the IS in SAS trials, so the response time is shorter, while the general movement pattern and the durations measured from the accelerometer data are preserved.

We have extracted different quantitative parameters from the EMG and accelerometer signals to examine trunk flexion in healthy human subjects, and to investigate the startle response in this trunk task. In future work, this study may be extended to evaluate trunk stability and the StartReact effect in patients with neurological injuries, such as SCI and stroke. Impaired trunk stability represents a serious problem that limits these patients’ mobility and daily life activities, so providing quantitative measures of trunk function may contribute to assessing patients’ condition and guiding rehabilitation interventions. On the other hand, the application of a SAS in neurologically impaired patients would allow us to investigate the descending tracts conveying the startle reaction in these patients, as well as the way in which a destabilizing unexpected stimulus can affect the compensation mechanisms of impaired trunk muscles.

Some of the limitations of this study are the relatively low number of SAS trials per subject, and that we explored the activation of trunk muscles in only one task. For technical reasons due to the limited number of channels, we recorded the EMG from only the left side of the body, so we could not measure the anticipatory body movements that would be produced in the right side of the body when lifting the left arm, which could be explored in future work. In addition, we could have examined other tasks or trunk movements in multiple directions to obtain more information about recruitment of trunk muscles for maintenance of stability in different situations. However, it would considerably increase the complexity and the time required for the experiment. Moreover, we preferred to focus on a simple task that could be extrapolated to individuals with varying degrees of disability, including paraplegic and tetraplegic patients, and populations with severely impaired trunk control.

In this work, we have described, documented, and quantified the patterns of muscle recruitment and accelerometer data that characterize the manoeuvre of trunk flexion in the context of a reaching task in able-bodied individuals. We have proposed different quantitative measures related to the execution of this movement, which could help to develop novel methods for evaluating trunk function. This is the first step to assess the changes that may occur in patients with neurological disorders linked to impairment of trunk stability control. The combination of neurophysiology measures and mobile health tools, such as smartphones, can provide valuable information for the assessment of trunk movements in an objective and cost-effective way.

## Data Availability

The dataset analysed during the current study is available from the corresponding authors on reasonable request.

## References

[1] Imai A, Kaneoka K, Okubo Y, Shiraki H (2014). Effects of two types of trunk exercises on balance and athletic performance in youth soccer players. Int. J. Sports Phys. Ther..

[2] Butcher SJ (2007). The effect of trunk stability training on vertical takeoff velocity. J. Orthop. Sports Phys. Ther..

[3] Massion J (1992). Movement, posture and equilibrium: Interaction and coordination. Prog. Neurobiol..

[4] Borghuis J, Hof AL, Lemmink KAPM (2008). The importance of sensory-motor control in providing core stability: Implications for measurement and training. Sports Med..

[5] Caronni A, Bolzoni F, Esposti R, Bruttini C, Cavallari P (2013). Accuracy of pointing movements relies upon a specific tuning between anticipatory postural adjustments and prime mover activation. Acta Physiol..

[6] Stamenkovic A, Stapley PJ (2016). Trunk muscles contribute as functional groups to directionality of reaching during stance. Exp. Brain Res..

[7] Bohannon RW, Cassidy D, Walsh S (1995). Trunk muscle strength is impaired multidirectionally after stroke. Clin. Rehabil..

[8] Rode G, Tiliket C, Boisson D (1997). Predominance of postural imbalance in left hemiparetic patients. Scand. J. Rehabil. Med..

[9] Tanaka S, Hachisuka K, Ogata H (1998). Muscle strength of trunk flexion-extension in post-stroke hemiplegic patients. Am. J. Phys. Med. Rehabil..

[10] Haruyama K, Kawakami M, Otsuka T (2017). Effect of core stability training on trunk function, standing balance, and mobility in stroke patients: A randomized controlled trial. Neurorehabil. Neural Repair.

[11] Forogh B (2017). The effect of repetitive transcranial magnetic stimulation on postural stability after acute stroke: A clinical trial. Basic Clin. Neurosci..

[12] Janssen-Potten YJM, Seelen HAM, Drukker J, Reulen JPH (2000). Chair configuration and balance control in persons with spinal cord injury. Arch. Phys. Med. Rehabil..

[13] Chen CL (2003). The relationship between sitting stability and functional performance in patients with paraplegia. Arch. Phys. Med. Rehabil..

[14] Gabison S (2014). Trunk strength and function using the multidirectional reach distance in individuals with non-traumatic spinal cord injury. J. Spinal Cord Med..

[15] Franchignoni FP, Tesio L, Ricupero C, Martino MT (1997). Trunk control test as an early predictor of stroke rehabilitation outcome. Stroke.

[16] Tasseel-Ponche S, Yelnik AP, Bonan IV (2015). Motor strategies of postural control after hemispheric stroke. Neurophysiol. Clin..

[17] Collin C, Wade D (1990). Assessing motor impairment after stroke: A pilot reliability study. J. Neurol. Neurosurg. Psychiatry.

[18] Quinzaños J, Villa AR, Flores AA, Pérez R (2014). Proposal and validation of a clinical trunk control test in individuals with spinal cord injury. Spinal Cord.

[19] Verheyden G (2004). The trunk impairment scale: A new tool to measure motor impairment of the trunk after stroke. Clin. Rehabil..

[20] Lynch SM, Leahy P, Barker SP (1998). Reliability of measurements obtained with a modified functional reach test in subjects with spinal cord injury. Phys. Ther..

[21] Katz-Leurer M, Fisher I, Neeb M, Schwartz I, Carmeli E (2009). Reliability and validity of the modified functional reach test at the sub-acute stage post-stroke. Disabil. Rehabil..

[22] Näf OB, Bauer CM, Zange C, Rast FM (2020). Validity and variability of center of pressure measures to quantify trunk control in stroke patients during quiet sitting and reaching tasks. Gait Posture.

[23] Milosevic M (2015). Trunk control impairment is responsible for postural instability during quiet sitting in individuals with cervical spinal cord injury. Clin. Biomech..

[24] Morasso P, Cherif A, Zenzeri J (2020). State-space intermittent feedback stabilization of a dual balancing task. Sci. Rep..

[25] Liao CF, Liaw LJ, Wang RY, Su FC, Hsu AT (2015). Electromyography of symmetrical trunk movements and trunk position sense in chronic stroke patients. J. Phys. Ther. Sci..

[26] Wang YJ (2016). Surface electromyography as a measure of trunk muscle activity in patients with spinal cord injury: A meta-Analytic review. J. Spinal Cord Med..

[27] Gauthier C (2012). Which trunk inclination directions best predict multidirectional-seated limits of stability among individuals with spinal cord injury?. J. Spinal Cord Med..

[28] Zhang, S., McCullagh, P., Nugent, C. & Zheng, H. Activity monitoring using a smart phone’s accelerometer with hierarchical classification. In *Proceedings—2010 6th International Conference on Intelligent Environments, IE 2010*, 158–163 (2010). 10.1109/IE.2010.36.

[29] Tacconi, C., Mellone, S. & Chiari, L. Smartphone-based applications for investigating falls and mobility. In *2011 5th International Conference on Pervasive Computing Technologies for Healthcare and Workshops, Pervasive Health 2011*, 258–261 (2011). 10.4108/icst.pervasivehealth.2011.246060.

[30] Ferrer-Lluis I, Castillo-Escario Y, Montserrat JM, Jane R (2020). Analysis of smartphone triaxial accelerometry for monitoring sleep-disordered breathing and sleep position at home. IEEE Access..

[31] Valls-Solé J (1995). Reaction time and acoustic startle in normal human subjects. Neurosci. Lett..

[32] Valls-Solé J, Rothwell JC, Goulart F, Cossu G, Muñoz E (1999). Patterned ballistic movements triggered by a startle in healthy humans. J. Physiol..

[33] Carlsen AN, Chua R, Inglis JT, Sanderson DJ, Franks IM (2004). Prepared movements are elicited early by startle. J. Mot. Behav..

[34] Carlsen AN, Chua R, Inglis JT, Sanderson DJ, Franks IM (2009). Differential effects of startle on reaction time for finger and arm movements. J. Neurophysiol..

[35] Siegmund GP, Inglis JT, Sanderson DJ (2001). Startle response of human neck muscles sculpted by readiness to perform ballistic head movements. J. Physiol..

[36] Queralt A, Valls-Solé J, Castellote JM (2008). The effects of a startle on the sit-to-stand manoeuvre. Exp. Brain Res..

[37] MacKinnon CD (2007). Preparation of anticipatory postural adjustments prior to stepping. J. Neurophysiol..

[38] Queralt A (2008). The effects of an auditory startle on obstacle avoidance during walking. J. Physiol..

[39] Castellote JM, Queralt A, Valls-Solé J (2012). Preparedness for landing after a self-initiated fall. J. Neurophysiol..

[40] Oude Nijhuis LB, Allum JHJ, Valls-Solé J, Overeem S, Bloem BR (2010). First trial postural reactions to unexpected balance disturbances: A comparison with the acoustic startle reaction. J. Neurophysiol..

[41] Kumru H, Valls-Solé J (2006). Excitability of the pathways mediating the startle reaction before execution of a voluntary movement. Exp. Brain Res..

[42] Maslovat D, Franks IM, Leguerrier A, Carlsen AN (2015). Responses to startling acoustic stimuli indicate that movement-related activation is constant prior to action: A replication with an alternate interpretation. Physiol. Rep..

[43] Carlsen AN, Maslovat D, Lam MY, Chua R, Franks IM (2011). Considerations for the use of a startling acoustic stimulus in studies of motor preparation in humans. Neurosci. Biobehav. Rev..

[44] Castillo-Escario, Y., Rodríguez-Cañón, M., García-Alías, G. & Jané, R. Onset Detection to study muscle activity in reaching and grasping movements in rats. In *Proc. Annu. Int. Conf. IEEE Eng. Med. Biol. Soc. (EMBC)*, 5113–5116 (2019). 10.1109/EMBC.2019.8857200 31947009

[45] Teager, H. M. & Teager, S. M. Evidence for nonlinear sound production mechanisms in the vocal tract. In *Speech Production and Speech Modelling,* 241–261 (Springer, 1990). 10.1007/978-94-009-2037-8_10.

[46] Li X, Zhou P, Aruin AS (2007). Teager-Kaiser energy operation of surface EMG improves muscle activity onset detection. Ann. Biomed. Eng..

[47] Carlsen AN, Maslovat D (2019). Startle and the StartReact effect: Physiological mechanisms. J. Clin. Neurophysiol..

[48] Stevenson AJT (2014). Cortical involvement in the StartReact effect. Neuroscience.

[49] Castellote JM, Valls-Solé J (2015). The StartReact effect in tasks requiring end-point accuracy. Clin. Neurophysiol..

